# 1,4,6,9-Tetra-*tert*-butyl-2,7-dioxa­tricyclo­[6.3.0.0^3,6^]deca-3,8-diene

**DOI:** 10.1107/S1600536813000147

**Published:** 2013-01-09

**Authors:** Ted S. Sorensen, S. M Humayaun Kabir, Masood Parvez

**Affiliations:** a Department of Chemistry, The University of Calgary, 2500 University Drive NW, Calgary, Alberta, Canada T2N 1N4

## Abstract

The title compound, C_24_H_40_O_2_, lies on an inversion center with a half-mol­ecule in the asymmetric unit. The central dioxane ring adopts a chair conformation. The four-membered ring is slightly puckered with a butterfly angle of 13.50 (14)°.

## Related literature

For the synthesis of the title compound, see: Rauk *et al.* (1995 ▶). For related structures, see: Masters *et al.* (1994 ▶); Bernassau *et al.* (1987 ▶).
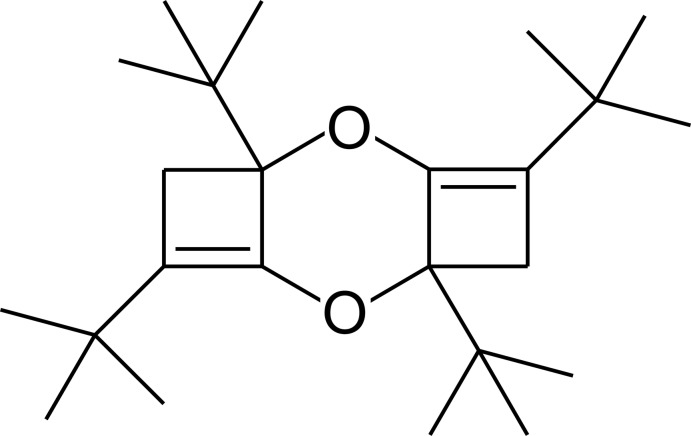


## Experimental

### 

#### Crystal data

C_24_H_40_O_2_
*M* *_r_* = 360.56
Triclinic, 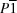

*a* = 5.843 (2) Å
*b* = 9.383 (3) Å
*c* = 10.126 (4) Å
α = 97.209 (12)°
β = 96.014 (13)°
γ = 100.703 (19)°
*V* = 536.5 (3) Å^3^
*Z* = 1
Mo *K*α radiation
μ = 0.07 mm^−1^
*T* = 170 K
0.20 × 0.15 × 0.05 mm

#### Data collection

Nonius APEXII CCD diffractometer
Absorption correction: multi-scan (*SORTAV*; Blessing, 1997 ▶) *T*_min_ = 0.987, *T*_max_ = 0.997
4499 measured reflections
2412 independent reflections
1957 reflections with *I* > 2σ(*I*)
*R*_int_ = 0.018

#### Refinement

*R*[*F* ^2^ > 2σ(*F* ^2^)] = 0.041
*wR*(*F* ^2^) = 0.104
*S* = 1.04
2412 reflections
124 parameters
H-atom parameters constrained
Δρ_max_ = 0.27 e Å^−3^
Δρ_min_ = −0.19 e Å^−3^

### 

Data collection: *COLLECT* (Hooft, 1998 ▶); cell refinement: *DENZO* (Otwinowski & Minor, 1997 ▶); data reduction: *SCALEPACK* (Otwinowski & Minor, 1997 ▶); program(s) used to solve structure: *SHELXS97* (Sheldrick, 2008 ▶); program(s) used to refine structure: *SHELXL97* (Sheldrick, 2008 ▶); molecular graphics: *ORTEP-3 for Windows* (Farrugia, 2012 ▶); software used to prepare material for publication: *SHELXL97*.

## Supplementary Material

Crystal structure: contains datablock(s) global, I. DOI: 10.1107/S1600536813000147/zl2528sup1.cif

Structure factors: contains datablock(s) I. DOI: 10.1107/S1600536813000147/zl2528Isup2.hkl

Additional supplementary materials:  crystallographic information; 3D view; checkCIF report

## Figures and Tables

**Fig. 1.**
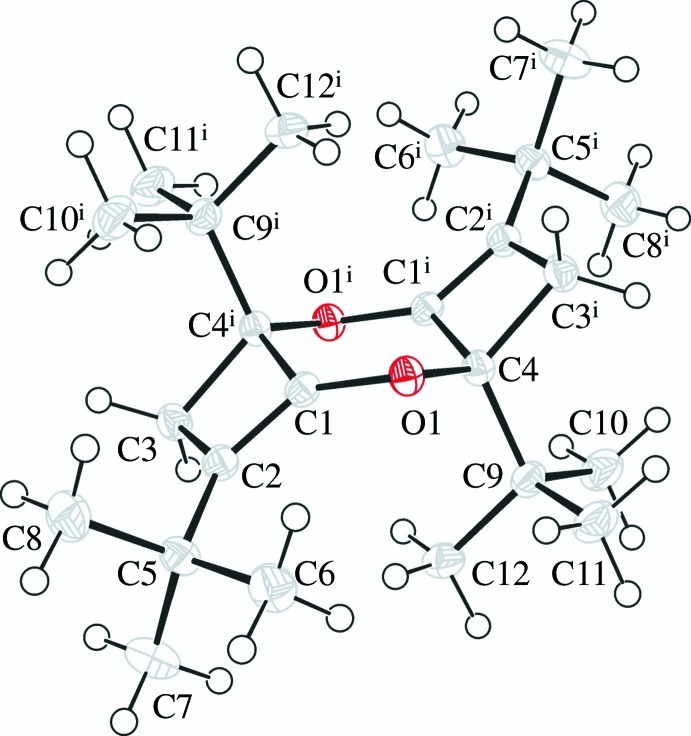
ORTEP drawing (Farrugia, 2012) of the title molecule with the displacement ellipsoids plotted at 50% probability level; H atoms are presented as small spheres of arbitrary radius. Symmetry code: i -x + 1, -y + 1, -z + 1.

**Fig. 2.**
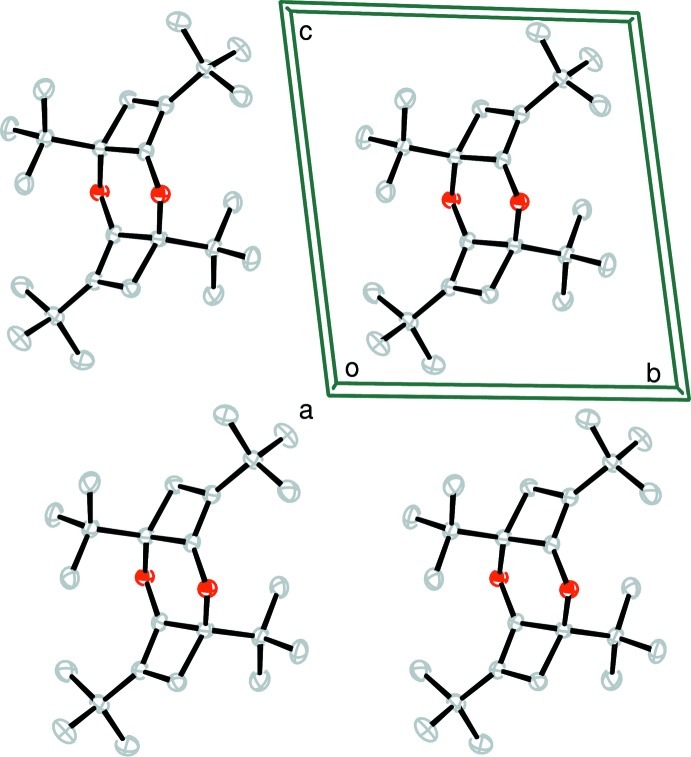
A view of the unit cell packing of the crystal structure of the title compound. H atoms were omitted for clarity.

## supplementary crystallographic information

### Comment

Our research on the preparation and structure investigations of simple bicyclo[1.1.0]butanones (Rauk *et al.* 1995) led to the synthesis of the title compound. The stereochemistry of the title compound was not known at that time. In the title compound (Fig. 1), the central dioxane ring adopts a chair conformation with puckering parameters: *Q* = 0.3934 (11) Å, θ = 0.74 (1)° and φ = 0.0°. The four membered ring (C1/C2/C3/C4^i^; ^i^ = -*x* + 1, -*y* + 1, -*z* + 1) is slightly puckered with the dihedral angle between mean planes C1/C2/C3 and C1/C3/C4^i^ being 13.50 (14)°. The molecular dimesions in the title compound agree very well with the corresponding molecular dimensions reported in closely related compounds (Masters *et al.*, 1994; Bernassau *et al.*, 1987). The crystal structure is devoid of any significant directional intermolecular interactions (Fig. 2).

### Experimental

The synthesis of the title compound has been reported earlier (Rauk *et al.*, 1995). Crystals suitable for crystallolgraphic studies were grown from pentane/CH_2_Cl_2_ (1:1).

### Refinement

Though the H-atoms were observable in the difference electron density maps they were included at geometrically idealized positions with C—H distances = 0.99 and 0.98 Å for methylene and methyl type H-atoms, respectively. The H-atoms were assigned *U*_iso_ = 1.2 times *U*_eq_(C).

### Crystal data

**Table:**

C_24_H_40_O_2_	*Z* = 1
*M**_r_* = 360.56	*F*(000) = 200
Triclinic, *P*1	*D*_x_ = 1.116 Mg m^−^^3^
Hall symbol: -P 1	Mo *K*α radiation, λ = 0.71073 Å
*a* = 5.843 (2) Å	Cell parameters from 2272 reflections
*b* = 9.383 (3) Å	θ = 1.0–27.5°
*c* = 10.126 (4) Å	µ = 0.07 mm^−^^1^
α = 97.209 (12)°	*T* = 170 K
β = 96.014 (13)°	Plate, colorless
γ = 100.703 (19)°	0.20 × 0.15 × 0.05 mm
*V* = 536.5 (3) Å^3^	

### Data collection

**Table:**

Nonius APEXII CCD diffractometer	2412 independent reflections
Radiation source: fine-focus sealed tube	1957 reflections with *I* > 2σ(*I*)
Graphite monochromator	*R*_int_ = 0.018
ω and φ scans	θ_max_ = 27.4°, θ_min_ = 4.1°
Absorption correction: multi-scan (*SORTAV*; Blessing, 1997)	*h* = −7→7
*T*_min_ = 0.987, *T*_max_ = 0.997	*k* = −12→12
4499 measured reflections	*l* = −13→13

### Refinement

**Table:**

Refinement on *F*^2^	Primary atom site location: structure-invariant direct methods
Least-squares matrix: full	Secondary atom site location: difference Fourier map
*R*[*F*^2^ > 2σ(*F*^2^)] = 0.041	Hydrogen site location: inferred from neighbouring sites
*wR*(*F*^2^) = 0.104	H-atom parameters constrained
*S* = 1.04	*w* = 1/[σ^2^(*F*_o_^2^) + (0.0415*P*)^2^ + 0.1563*P*] where *P* = (*F*_o_^2^ + 2*F*_c_^2^)/3
2412 reflections	(Δ/σ)_max_ < 0.001
124 parameters	Δρ_max_ = 0.27 e Å^−^^3^
0 restraints	Δρ_min_ = −0.19 e Å^−^^3^

### Special details

**Table:**

Geometry. All e.s.d.'s (except the e.s.d. in the dihedral angle between two l.s. planes) are estimated using the full covariance matrix. The cell e.s.d.'s are taken into account individually in the estimation of e.s.d.'s in distances, angles and torsion angles; correlations between e.s.d.'s in cell parameters are only used when they are defined by crystal symmetry. An approximate (isotropic) treatment of cell e.s.d.'s is used for estimating e.s.d.'s involving l.s. planes.
Refinement. Refinement of *F*^2^ against ALL reflections. The weighted *R*-factor *wR* and goodness of fit *S* are based on *F*^2^, conventional *R*-factors *R* are based on *F*, with *F* set to zero for negative *F*^2^. The threshold expression of *F*^2^ > σ(*F*^2^) is used only for calculating *R*-factors(gt) *etc*. and is not relevant to the choice of reflections for refinement. *R*-factors based on *F*^2^ are statistically about twice as large as those based on *F*, and *R*- factors based on ALL data will be even larger.

### Fractional atomic coordinates and isotropic or equivalent isotropic displacement parameters (Å^2^)

**Table:**

	*x*	*y*	*z*	*U*_iso_*/*U*_eq_	
O1	0.25855 (13)	0.40276 (8)	0.50016 (8)	0.0194 (2)	
C1	0.36790 (19)	0.43605 (12)	0.39096 (11)	0.0183 (2)	
C2	0.38127 (19)	0.36693 (12)	0.26899 (11)	0.0195 (2)	
C3	0.6042 (2)	0.47693 (13)	0.25541 (12)	0.0225 (3)	
H3A	0.5931	0.5253	0.1742	0.027*	
H3B	0.7506	0.4384	0.2666	0.027*	
C4	0.44599 (19)	0.43136 (12)	0.61504 (11)	0.0184 (2)	
C5	0.2453 (2)	0.23533 (13)	0.17350 (12)	0.0226 (3)	
C6	0.0667 (2)	0.14183 (14)	0.24394 (13)	0.0304 (3)	
H6A	−0.0295	0.0617	0.1785	0.037*	
H6B	−0.0346	0.2029	0.2839	0.037*	
H6C	0.1499	0.1010	0.3146	0.037*	
C7	0.4145 (2)	0.14319 (15)	0.11795 (14)	0.0343 (3)	
H7A	0.3251	0.0586	0.0549	0.041*	
H7B	0.5003	0.1089	0.1921	0.041*	
H7C	0.5263	0.2032	0.0714	0.041*	
C8	0.1150 (2)	0.28994 (15)	0.05637 (13)	0.0335 (3)	
H8A	0.0317	0.2058	−0.0093	0.040*	
H8B	0.2283	0.3532	0.0133	0.040*	
H8C	0.0017	0.3457	0.0903	0.040*	
C9	0.5229 (2)	0.28551 (12)	0.63873 (12)	0.0219 (3)	
C10	0.7097 (2)	0.30967 (15)	0.76230 (14)	0.0325 (3)	
H10A	0.7518	0.2159	0.7764	0.039*	
H10B	0.6468	0.3501	0.8416	0.039*	
H10C	0.8497	0.3785	0.7477	0.039*	
C11	0.3063 (2)	0.17552 (14)	0.66121 (14)	0.0306 (3)	
H11A	0.3515	0.0826	0.6755	0.037*	
H11B	0.1872	0.1588	0.5822	0.037*	
H11C	0.2418	0.2149	0.7403	0.037*	
C12	0.6320 (2)	0.22114 (13)	0.51932 (13)	0.0273 (3)	
H12A	0.6876	0.1332	0.5403	0.033*	
H12B	0.7646	0.2940	0.5018	0.033*	
H12C	0.5136	0.1952	0.4396	0.033*	

### Atomic displacement parameters (Å^2^)

**Table:**

	*U*^11^	*U*^22^	*U*^33^	*U*^12^	*U*^13^	*U*^23^
O1	0.0169 (4)	0.0215 (4)	0.0192 (4)	0.0009 (3)	0.0034 (3)	0.0036 (3)
C1	0.0168 (5)	0.0176 (5)	0.0209 (6)	0.0029 (4)	0.0030 (4)	0.0050 (4)
C2	0.0194 (5)	0.0186 (5)	0.0202 (6)	0.0028 (4)	0.0029 (4)	0.0033 (4)
C3	0.0249 (6)	0.0216 (6)	0.0204 (6)	0.0019 (5)	0.0060 (5)	0.0022 (5)
C4	0.0179 (5)	0.0192 (5)	0.0173 (6)	0.0016 (4)	0.0023 (4)	0.0026 (4)
C5	0.0223 (6)	0.0224 (6)	0.0212 (6)	0.0024 (5)	0.0022 (5)	−0.0007 (5)
C6	0.0286 (6)	0.0280 (7)	0.0292 (7)	−0.0041 (5)	0.0021 (5)	−0.0005 (5)
C7	0.0310 (7)	0.0306 (7)	0.0373 (8)	0.0061 (6)	0.0040 (6)	−0.0098 (6)
C8	0.0367 (7)	0.0359 (7)	0.0244 (7)	0.0045 (6)	−0.0033 (5)	0.0010 (5)
C9	0.0241 (6)	0.0188 (6)	0.0230 (6)	0.0028 (4)	0.0034 (5)	0.0059 (5)
C10	0.0387 (7)	0.0283 (7)	0.0307 (7)	0.0095 (6)	−0.0036 (6)	0.0081 (5)
C11	0.0340 (7)	0.0208 (6)	0.0382 (7)	0.0020 (5)	0.0091 (6)	0.0098 (5)
C12	0.0305 (6)	0.0226 (6)	0.0312 (7)	0.0106 (5)	0.0054 (5)	0.0046 (5)

### Geometric parameters (Å, º)

**Table:**

O1—C1	1.3744 (14)	C7—H7A	0.9800
O1—C4	1.4733 (14)	C7—H7B	0.9800
C1—C2	1.3380 (16)	C7—H7C	0.9800
C1—C4^i^	1.5048 (15)	C8—H8A	0.9800
C2—C5	1.5053 (16)	C8—H8B	0.9800
C2—C3	1.5344 (16)	C8—H8C	0.9800
C3—C4^i^	1.5615 (16)	C9—C11	1.5338 (17)
C3—H3A	0.9900	C9—C12	1.5347 (17)
C3—H3B	0.9900	C9—C10	1.5361 (18)
C4—C1^i^	1.5048 (15)	C10—H10A	0.9800
C4—C9	1.5557 (16)	C10—H10B	0.9800
C4—C3^i^	1.5615 (16)	C10—H10C	0.9800
C5—C6	1.5299 (17)	C11—H11A	0.9800
C5—C7	1.5340 (17)	C11—H11B	0.9800
C5—C8	1.5354 (18)	C11—H11C	0.9800
C6—H6A	0.9800	C12—H12A	0.9800
C6—H6B	0.9800	C12—H12B	0.9800
C6—H6C	0.9800	C12—H12C	0.9800
			
C1—O1—C4	105.98 (8)	C5—C7—H7C	109.5
C2—C1—O1	136.58 (10)	H7A—C7—H7C	109.5
C2—C1—C4^i^	95.84 (9)	H7B—C7—H7C	109.5
O1—C1—C4^i^	127.01 (10)	C5—C8—H8A	109.5
C1—C2—C5	137.73 (11)	C5—C8—H8B	109.5
C1—C2—C3	91.75 (9)	H8A—C8—H8B	109.5
C5—C2—C3	130.52 (10)	C5—C8—H8C	109.5
C2—C3—C4^i^	86.07 (8)	H8A—C8—H8C	109.5
C2—C3—H3A	114.3	H8B—C8—H8C	109.5
C4^i^—C3—H3A	114.3	C11—C9—C12	109.70 (10)
C2—C3—H3B	114.3	C11—C9—C10	109.15 (10)
C4^i^—C3—H3B	114.3	C12—C9—C10	106.61 (10)
H3A—C3—H3B	111.5	C11—C9—C4	108.43 (10)
O1—C4—C1^i^	112.31 (9)	C12—C9—C4	111.81 (10)
O1—C4—C9	109.61 (9)	C10—C9—C4	111.11 (10)
C1^i^—C4—C9	118.82 (9)	C9—C10—H10A	109.5
O1—C4—C3^i^	115.51 (9)	C9—C10—H10B	109.5
C1^i^—C4—C3^i^	84.71 (8)	H10A—C10—H10B	109.5
C9—C4—C3^i^	114.21 (9)	C9—C10—H10C	109.5
C2—C5—C6	110.63 (10)	H10A—C10—H10C	109.5
C2—C5—C7	109.91 (10)	H10B—C10—H10C	109.5
C6—C5—C7	109.95 (11)	C9—C11—H11A	109.5
C2—C5—C8	108.22 (10)	C9—C11—H11B	109.5
C6—C5—C8	109.26 (11)	H11A—C11—H11B	109.5
C7—C5—C8	108.82 (11)	C9—C11—H11C	109.5
C5—C6—H6A	109.5	H11A—C11—H11C	109.5
C5—C6—H6B	109.5	H11B—C11—H11C	109.5
H6A—C6—H6B	109.5	C9—C12—H12A	109.5
C5—C6—H6C	109.5	C9—C12—H12B	109.5
H6A—C6—H6C	109.5	H12A—C12—H12B	109.5
H6B—C6—H6C	109.5	C9—C12—H12C	109.5
C5—C7—H7A	109.5	H12A—C12—H12C	109.5
C5—C7—H7B	109.5	H12B—C12—H12C	109.5
H7A—C7—H7B	109.5		
			
C4—O1—C1—C2	124.33 (14)	C1—C2—C5—C7	−133.88 (15)
C4—O1—C1—C4^i^	−44.73 (14)	C3—C2—C5—C7	46.30 (16)
O1—C1—C2—C5	19.1 (2)	C1—C2—C5—C8	107.40 (16)
C4^i^—C1—C2—C5	−169.70 (13)	C3—C2—C5—C8	−72.42 (15)
O1—C1—C2—C3	−161.07 (13)	O1—C4—C9—C11	−57.21 (12)
C4^i^—C1—C2—C3	10.17 (9)	C1^i^—C4—C9—C11	171.79 (10)
C1—C2—C3—C4^i^	−9.77 (9)	C3^i^—C4—C9—C11	74.21 (12)
C5—C2—C3—C4^i^	170.11 (12)	O1—C4—C9—C12	63.86 (12)
C1—O1—C4—C1^i^	37.41 (12)	C1^i^—C4—C9—C12	−67.14 (13)
C1—O1—C4—C9	−96.97 (10)	C3^i^—C4—C9—C12	−164.73 (9)
C1—O1—C4—C3^i^	132.31 (10)	O1—C4—C9—C10	−177.16 (9)
C1—C2—C5—C6	−12.26 (19)	C1^i^—C4—C9—C10	51.84 (14)
C3—C2—C5—C6	167.92 (11)	C3^i^—C4—C9—C10	−45.74 (13)

Symmetry code: (i) −*x*+1, −*y*+1, −*z*+1.
